# Role of low protein diet in management of different stages of chronic kidney disease - practical aspects

**DOI:** 10.1186/s12882-016-0360-1

**Published:** 2016-10-21

**Authors:** Bharat V. Shah, Zamurrud M. Patel

**Affiliations:** Institute of Renal Sciences, Global Hospital, Parel, Mumbai, 400012 India

**Keywords:** Low protein diet, Chronic kidney disease, Pre-ESRD, Very low protein diet, Ketoanalogues, Practical aspects

## Abstract

**Background:**

Chronic kidney disease (CKD) is a worldwide public health problem and more so in India. With limited availability and high cost of therapy, barely 10 % of patients with incident end stage renal disease (ESRD) cases get treatment in India. Therefore, all possible efforts should be made to retard progression of CKD. This article reviews the role of low protein diet (LPD) in management of CKD subjects and suggests how to apply it in clinical practice.

**Discussion:**

The role of LPD in retarding progression of CKD is well established in animal experimental studies. However, its role in human subjects with CKD is perceived to be controversial based on the modification of diet in renal disease (MDRD) study. We believe that beneficial effect of LPD could not be appreciated due to shorter duration of follow-up in the MDRD study. Had the study been continued longer, it may have been possible to appreciate beneficial effect of LPD. It is our contention that in all cases of CKD that are slowly progressive, LPD can significantly retard progression of CKD and delay the need for renal replacement therapy (RRT). To be able to apply LPD for a long period, it is important to prescribe LPD at earlier stages (1,2,3) of CKD and not at late stage as recommended by KDIGO guidelines. Many clinicians are concerned about worsening nutritional status and hence reluctant to prescribe LPD. This actually is true for patients with advanced CKD in whom there is spontaneous decrease in calorie and protein intake. In our experience, nutritional status of patients in early stages (1,2,3) of CKD is as good as that of healthy subjects. Prescribing LPD at an early stage is unlikely to worsen status.

**Summary:**

The role of LPD in retarding progression of CKD is well established in animal experimental studies. Even in human subjects, there is enough evidence to suggest that LPD retards progression of CKD in carefully selected subjects. It should be prescribed to those with good appetite, good nutritional status and a slowly progressive CKD at an early stage (stage 1,2,3). It may also be prescribed at stage 4 & 5 of CKD if the appetite and nutritional status are good.

## Background

Chronic kidney disease (CKD) is a worldwide public health problem. This problem is even more in socioeconomically deprived countries because lower income and social deprivation are associated with higher incidence of macroalbuminuria, reduced GFR, progressive kidney function loss and end stage renal disease (ESRD) [1–3]. Therefore, the problem of CKD is likely to be higher in India and other socioeconomically deprived countries than in the affluent western countries. The problem of CKD in India is likely to be higher also because of rising burden of diabetes and hypertension [4, 5].

In the absence of a Govt. set up national renal registry, the exact disease burden of CKD and ESRD in the Indian population is unknown. However, an Indian population-based study determined the crude ESRD incidence rates at 151 per million population [6]. This however seems to be an underestimate for reasons cited above.

With limited availability and high cost of therapy, barely 10 % of patients with incident ESRD cases get treatment in India [7]. Therefore, major emphasis should be on early detection of CKD and application of all possible measures to retard progression of CKD. The important role of blood pressure control in retarding progression of CKD is well established. However, the role of low protein diet (LPD) remains controversial. This article reviews the role of LPD in management of CKD subjects and suggests how to apply it in clinical practice.

## Discussion

### Role of LPD

**Fig. 1:**
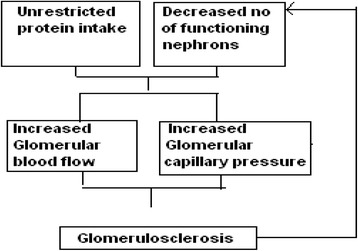
Hypothesis proposed by Brenner et al. [13]. Unrestricted protein intake in the face of decreased number of functioning nephrons leads to increase in glomerular capillary flow and glomerular capillary pressure. These hemodynamic changes lead to glomerulosclerosis. This results in further reduction in functioning nephrons and setting up of a vicious cycle which culminates in end stage renal disease

Studies in animals have clearly shown that high protein intake relative to functioning renal mass contributes to progressive decline in kidney function [8–12]. Based on observations in animal experimental studies, Brenner et al. [13] proposed a hypothesis. They proposed that when the functioning renal mass is reduced, hemodynamic changes develop in the remnant nephrons. These changes, which partially offset the loss of function that would result, are compensatory or adaptive. It is these adaptive changes that contribute to progressive deterioration in renal function (Fig. 1). Restricting dietary protein early in the course of renal disease can minimize the adaptive changes and thereby retard progressive deterioration in renal function.

With Brenner’s hypothesis, there was a resurgence of interest in LPD. Before the hypothesis, LPD was practiced as suggested by Giordano and Giovannetti [14] mainly to mitigate uremic symptoms in advanced CKD. After the hypothesis, the interest was to study the effect of LPD in retarding progression of CKD in human subjects. These studies did suggest a beneficial effect [15, 16]. However, there were two major limitations of these studies: 1) they used creatinine as a marker of kidney function (which we now know is not an ideal marker of kidney function) and 2) they used 1/creatinine vs. time plot with patient as his own control, as proposed by Mitch et al. [17] to monitor the rate of progression of CKD, which Shah and Levey [18] have shown is not appropriate. The use of these inappropriate parameters cast doubt on interpretations of the earlier studies of LPD.

The limitations of earlier studies were eliminated in the modification of Diet in Renal Disease (MDRD) Study [19] which used renal clearance of Iothalamate to assess the GFR and GFR vs. time plot to monitor rate of decline in renal function in comparable groups of patients. In this study, 585 patients were included in study A & 255 patients were included in study B. Study A included patients with glomerular filtration rate (GFR) 25 to 55 ml/min and they were prescribed usual (1.3 gm/kg/day) or low protein (0.58 gm/kg/day). Study B included patients with GFR 13 to 24 ml/min. and they were prescribed low protein diet (0.58 gm/kg/day) or very low protein diet (0.28gm/kg/day) supplemented with ketoanalogs (KA). In both Study A & B, diabetics were excluded. The conclusion of the study was that that there was some slowing in the rate of decline in GFR in study A and no significant difference in the rate of decline in GFR in study B.

Around the time, MDRD study was published, we were looking at dietary protein intake in our stable patients with CKD stage 4 and 5. We observed that most of our subjects were predominantly vegetarians and their mean ± 1 SD protein intake was low (0.65 ± 0.15) gm/kg/day even when not prescribed any restriction [20]. A similar observation was made by Ikizler et. al. [21]. Considering such low protein intake, we thought that there was limited scope for prescribing any dietary protein restriction to Indian subjects with CKD, particularly vegetarians even if there was any benefit of LPD.

In 2002, Kher [22], in a nephrology forum discussed about huge burden of ESRD in India and limited availability and affordability of RRT. This prompted us to review the role of LPD in preventing and retarding progression of CKD to ESRD.

We reviewed the MDRD study and realized that its major limitation was the duration of the study. In any slowly progressive condition, a longer follow-up is required to appreciate the effect of any therapeutic intervention. For example, in the Diabetes Control and Complication trial (DCCT), there was no difference in development of microalbuminuria or clinical albuminuria at 3 to 4 years in the groups treated conventionally or with intensive insulin therapy. However, on long-term follow-up, a significantly lower percentage of patients developed microalbuminuria and clinical albuminuria in the group treated intensively with insulin [23].

**Fig. 2:**
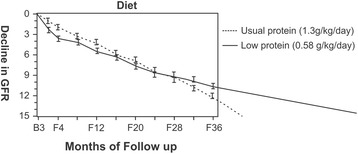
A longer duration of study required to appreciate beneficial effect of LPD. The graph shows rate of decline in GFR in patients on usual protein (1.3 g/kg/day) and in those on low protein (0.58 g/kg/dy). Because of initial rapid decline in GFR which was hemodynamically mediated, although subsequent rate of decline was slower in subjects prescribed a low protein diet (*solid line*), the absolute decrease in GFR was not significantly different when compared to subjects allowed usual protein diet over a follow-up period of 36 months (F36). If the study had been continued further, it would have been possible to see the beneficial effect of low protein diet compared to usual protein diet as shown by extrapolated lines

In the MDRD study A patients, there was a rapid decline in kidney function in subjects with low protein diet for the first 4 months. This was hemodynamically mediated. After 4 months the rate of decline in kidney functions in patients with protein restriction was slower than in those on usual protein diet. Had the study been continued further, it would have been possible to appreciate beneficial effect of dietary protein restriction [24]. Figure 2 shows potential benefit of dietary protein restriction on longer follow-up.

Even otherwise, secondary analysis of the MDRD study suggested that dietary protein restriction was beneficial [25, 26].

Observing that a longer period of treatment can show beneficial effect of LPD in subjects with CKD and that secondary analysis of MDRD study did show beneficial effect of LPD, we realized that it is important to advise dietary protein restriction at an early stage of CKD and to those with slowly progressive CKD. Also some Indian studies reported beneficial effect of LPD.

Prakash et al [27] conducted a randomized. Double-blind, placebo controlled trial to evaluate efficacy of VLPD supplemented with KA in patients with CKD. Thirty-four patients were randomized to 2 comparable groups in terms of age, sex distribution, etiology of CKD, blood pressure control, use of angiotensin converting enzyme inhibitors, GFR and body mass index (BMI). Subjects randomly received either 0.6 gm/kg/day protein plus placebo (*n* = 16) or 0.3 gm/kg/day protein plus 1 tablet/5 kg of KA (Ketosteril;Fresenius Kabi, Germany) for 9 months. The mean GFR at baseline in the KA group and control group was 28.1 + 8.8 and 28.6 + 17.6 ml/min/1.73 m2 respectively. At the end of the study it was 27.6 + 10.1 and 22.5 + 15.9 ml/min/1.73 m2 respectively. Thus there was a significant drop in GFR in the control group compared to KA group. In both groups there was no significant change in the BMI after the study.

Subhramanyam et al. [28] assessed the effect of low protein diet in 178 adult patients with CKD Stages 3–5 (predialysis) for 1 year. Based on affordability of costly KA, Low-protein diet (0.6 g/kg) supplemented with one KA tablet for every 10 kg body weight (BW) was prescribed to 122 patients (sLPD group) and very low protein diet supplemented with one KA tablet for every 5 kg BW was prescribed to 56 patients s(VLPD group). In the sLPD group, the blood urea level decreased from 85.38 ± 4.45 to 76.90 ± 42.90 mg/dl (*p* < 0.05) after 12 months. The 24-h urinary creatinine clearance (CrCl) improved from 24.59 ± 16.13 ml/min to 29.45 ± 28.16 mL/min after 12 months. In the sVLPD group, the blood urea level which was 98.38 ± 42.97 mg/dl at baseline marginally increased to 102.74 ± 45.98 mg/dL (*p* > 0.05) at the end of 1 year. The CrCl improved from 17.25 ± 9.25 ml/min at baseline to 18.24 ± 12.12 mL/min at the end of 1 year, but this increase was not statistically significant. There was a decrease in urinary protein excretion and improvement in metabolic status, and nutrition in both groups. In this study it is difficult to understand improvement in CrCl and better outcome with sLPD compared to sVLPD.

**Fig. 3:**
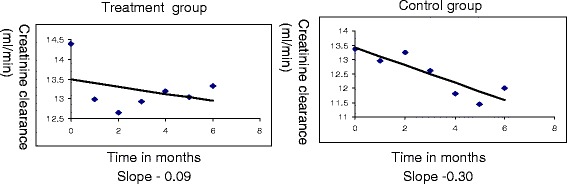
Efficacy of VLPD + KA on rate of decline in creatinine clearance in patients with slowly progressive CKD. The slope of creatinine clearance vs. time was −0.09 ml/min/month in patients prescribed 0.3 g/kg/day mixed protein supplemented with ketoanalogues (treatment group) while it was −0.3 in the patients allowed to continue regular diet (control group). One can notice that in the treatment group there was an initial rapid drop in creatinine clearance (possibly hemodynamically mediated). Thereafter, creatinine clearance stabilized. On the other hand, there was a progressive decline in creatinine clearance in the control group

We also studied efficacy and safety of KA supplemented very low protein diet in patients with CKD. The study included 29 stable stage V (non-diabetic) patients with slowly progressive CKD. Fourteen agreed to treatment with KA (treatment group) and 15 did not agree (control group). The patients in both groups were comparable as regards age, sex, dietary habits, degree of renal dysfunction and degree of proteinuria. Patients in the treatment group were prescribed 0.3 g/kg/day mixed protein supplemented with KA (ketosteril 1 tablet/5 kg body weight) while patients in the control group were allowed to continue regular diet. Renal function was monitored from 24 h urinary creatinine clearance and protein intake was monitored from urea nitrogen appearance (UNA) i.e. 6.25(24 h urinary urea nitrogen + 0.031xweight in kg). All were followed monthly for 6 months. The rate of decline in creatinine clearance was 0.09 ml/min/month in the treatment group while it was 0.3 ml/min/month in the control group (Fig. 3).

The concern about prescribing dietary protein restriction is that it is difficult to follow and that it increases the risk of malnutrition. As regards, difficulty in following LPD, one needs a good dietician who can help patients adjust to protein restricted diet. We also conduct a kidney workshop every week educating patients with CKD. In the workshop we educate patients about progressive nature of CKD and limited treatment options when kidneys fail. This education also helps patients work hard to follow every possible measure including dietary modification to retard progression of CKD.

**Fig. 4:**
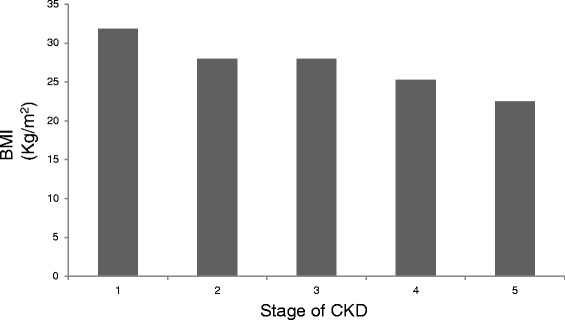
Body-mass index (kg/m^2^) of patients in different stages of CKD. The nutritional status of patients remains good until stage 3 of CKD. It tends to decline only in the late stages of CKD (stage 4 & 5)

The risk of malnutrition is not true if LPD is instituted at an early stage when the appetite and nutritional status is good. We looked at body-mass index [BMI] of 560 patients in different stages of CKD (Fig. 4). The mean BMI (kg/m2) of patients with stage 1 CKD was 32; stage 2 CKD, 28; stage 3 CKD, 28; stage 4 CKD, 25 and stage 5 CKD, 22.5. This shows that nutritional status of patients as judged from BMI is good until stage 3 of CKD and tends to drop thereafter. This has important clinical implication. Dietary modification should be prescribed in early stages of CKD and not in late stage (stage 4 & 5) as practiced by many and suggested by KDIGO [29].

In summary there is enough evidence to suggest that LPD retards the rate of progression of CKD. It should be implemented at an early stage of CKD and in those with slowly progressive disease to be able to appreciate its beneficial effects. We do not agree with KDIGO recommendations of lowering protein intake to 0.8 g/kg/day only when GFR <30 ml/min/1.73 m2 (GFR categories G4-G5), In our opinion, LPD (0.6 to 0.8 g/kg/day) should be prescribed to those with slowly progressive CKD at an early stage (1, 2 and 3) and KA supplemented very low protein diet should be prescribed to those with good appetite, good nutritional status and slowly progressive CKD at an advanced stage (4 and 5).

### Practical aspects of LPD

At the outset, one needs to decide who should be subjected to LPD. Not all patients benefit from LPD. In fact, in some it may prove to be detrimental. Therefore, prescription of dietary protein will vary from case to case.

**Fig. 5:**
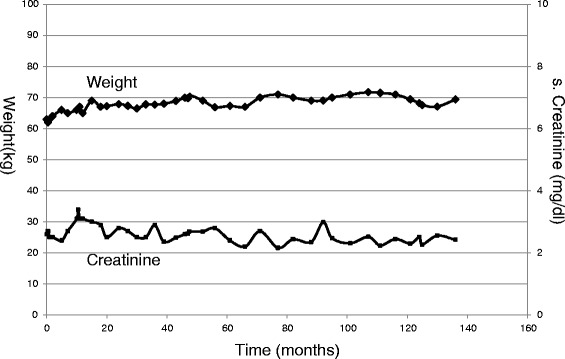
Example of a vegetarian patient with stable renal function without dietary modification. Serial weight and creatinine values in a 62 years old vegetarian female with no edema and non-proteinuric kidney disease without any dietary modification prescribed. The serum creatinine has remained stable for last 12 years

In practice, one does come across patients with CKD who maintain stable renal function without any dietary modification. Such patients need not be prescribed any LPD. Figure 5 shows an example of a patient (vegetarian) who has maintained stable renal function and stable nutritional status for more than 10 years. Her current weight is 71 kg (62 kg 12 years back) and serum albumin is 4 g/dl. Intermittent assessment of her protein intake from urea nitrogen appearance (UNA) has shown her protein intake to be 0.6 to 0.8 g/kg/day without any prescription of LPD. It is likely that this low protein intake has helped her remain very stable. One may argue that creatinine is not a good marker of renal function. That is true only in the context of an edematous patient or a patient changing diet and losing muscle mass. In absence of that, as in our case, creatinine is still the simplest and reliable marker of kidney function.

**Fig. 6:**
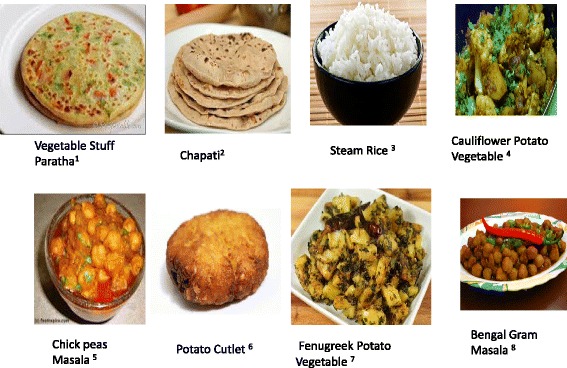
North Indian cuisine

**Fig. 7:**
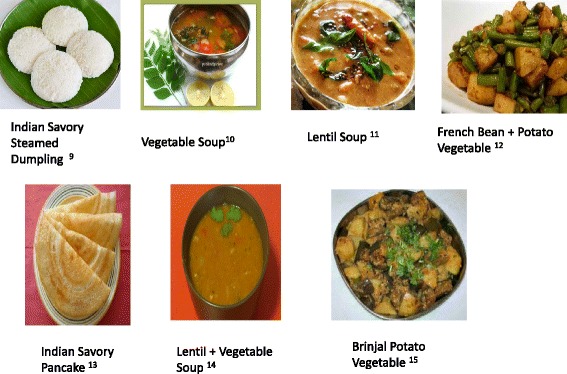
South Indian cuisine

In patients with slowly progressive CKD at an early stage (1,2,3) if protein intake is > 0.8 g/kg/day, we prescribe a LPD. Table 1 and Figs. 6 and 7 show standard north and south Indian cuisine plan providing 33 kcal/kg/day and 0.6 g/kg/day protein for a patient weighing 60 kg.

**Table 1:** A Standard North Indian & South Indian Cuisine Plan for LPD (0.6gm/kg/day) for a 60 kg patient

	North Indian menu	South Indian menu
B’fast:	Tea 1 Cup (Cow Milk 50 ml)	Coffee 1 Cup (Cow Milk 50 ml)
	Cereal 30 gm (e.g. Vegetable Stuffed Wheat Paratha^1^)	Cereal 30 gm (e.g. Idli^9^)
	Vegetable 50 gm	Lentil 15 gm
	Yoghurt (Cow Milk based) 50 gm	Vegetable 100 gm (e.g. Vegetable Rasam^10^)
Mid Morning:	Fruit 1 (100 gm)	Fruit 1 (100 gm)
Lunch:	Cereal + Starch Flour 150 gm (e.g. Wheat Chapati ^2^ + Boil Rice^3^)	Cereal 90 gm (e.g. Boil Rice)
	Vegetable 200 gm (e.g. Gobi Aloo Vegetable^4^)	Lentil 30 gm (e.g. Vegetable Sambhar^11^)
	Lentil 15 gm (e.g. Chole Masala^5^)	Vegetable 200 gm (e.g. Bean Aloo Vegetable^12^)
	Yoghurt (Cow Milk based) 50 gm	Yoghurt (Cow Milk based) 50 gm
Teatime:	Tea 1 Cup (Cow Milk 50 ml)	Coffee 1 Cup (Cow Milk 50 ml)
	Starch Vegetable 100 gm (e.g. Aloo Tikki^6^)	Cereal 30 gm (e.g. Dosa^13^)
Mid-Evening:	Fruit 1 (100 gm)	Fruit 1 (100 gm)
Dinner:	Cereal + Starch Flour 150 gm (e.g. Wheat Chapati + Boil Rice)	Cereal 90 gm (e.g. Boil Rice)
	Vegetable 200 gm (e.g. Methi Aloo Vegetable^7^)	Lentil 30 gm (e.g. Dal Rasam^14^)
	Lentil 15 gm (e.g. Chana Masala^8^)	Vegetable 150 gm (e.g. Brinjal Potato Vegetable^15^)
	Yoghurt (Cow Milk based) 50 gm	Yoghurt (Cow Milk based) 50 gm
Note:	To use Oil and Ghee 6 tsp/day	
	See - Legend Table	

English names: 1 = Indian Bread Stuffed with vegetables, 2 = Indian Bread, 3 = Steam Rice, 4 = Cauliflower Potato Vegetable, 5 = Chickpeas Vegetable, 6 = Potato Cutlet 7 = Fenugreek Potato Vegetable, 8 = Bengal Gram Vegetable, 9 = Indian Savory Steamed Dumpling, 10 = Vegetable Soup, 11 = Lentil + Vegetable Soup, 12 = French Potato Vegetable, 13 = Indian Savory Pancake, 14 = Lentil + Vegetable Soup, 15 = Brinjal Potato Vegetable

**Fig. 8:**
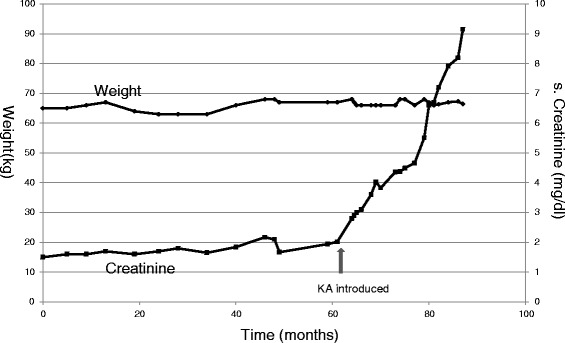
Example to show lack of benefit of dietary modification when kidney function is deteriorating rapidly. Serial weight and creatinine values in a 52 years old male with hypertensive nephrosclerosis. He maintained stable creatinine for many years on regular diet. He then resorted to alternative therapy. Following that, his creatinine started rising rapidly. Very low protein diet supplemented with KA was started but had no significant impact

If the kidney disease is rapidly progressive, LPD is not of much help. Figure 8 shows an example of a case of rapidly worsening kidney function. This 52 years old patient had CKD due to hypertensive nephrosclerosis. He was stable for many years on regular diet. He then resorted to alternative therapy. Following that, his renal function started deteriorating. Very low protein diet (0.3 g/kg/day) supplemented with 13 tablets of KA (for his edema free weight of 63 kg) was prescribed when creatinine began to rise rapidly. During this period, his 24 h urinary urea nitrogen was 3.17 g (0.47 g contributed by 13 tablets of KA). Adding extra-renal nitrogen loss (assumed to be 0.031 g/kg) his total nitrogen loss came to 5.12 g/day. Thus his protein intake was 32 g/day (0.48 g/kg/day). This protein restriction however had no significant impact on the rate of decline in his kidney function. He eventually underwent preemptive kidney transplant with mother as donor.

**Fig. 9:**
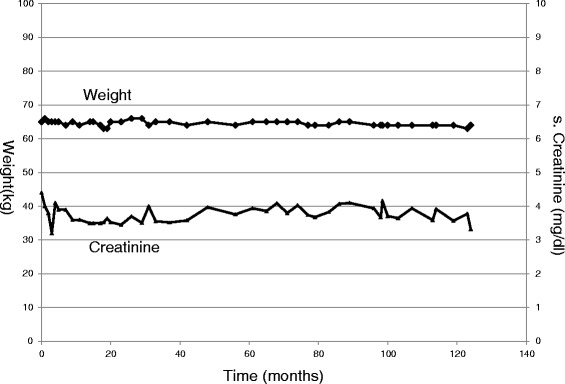
Example to show beneficial effect of LPD with slowly progressive CKD. Serial weight and creatinine values in a 83 years old physician who was detected to have slowly progressive CKD in 2006. He was prescribed VLPD supplemented with KA. He has maintained a very stable creatinine for 10 years

**Fig. 10:**
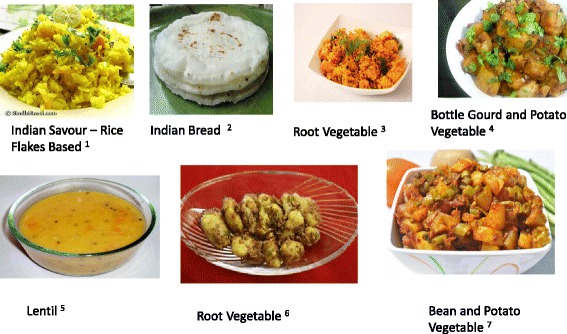
Sample menu of VLPD

When kidney disease is slowly progressive, LPD is likely to be helpful. Figure 9 shows example of a 83 years old physician who was detected to have slowly progressive CKD in 2006. He was prescribed VLPD (Table 2 and Fig. 10) supplemented with KA (1 tablet/5 kg). He has maintained a very stable creatinine and nutritional status for 10 years. His current weight is 65 kg, BMI 23.35 kg/m2 and serum albumin 4.6 g/dl. We are not sure if he would have remained stable without LPD which was advised only when he had 3 consecutive values of creatinine showing a rising trend. His protein intake (dietary plus supplemented KA) as judged from UNA remains about 0.48 to 0.5 g/kg/day.

**Table 2:** A Standard VLPD (0.3gm/kg/day) plan for a Patient weighing 70 kg

B’fast:	Tea 1 Cup (Cow Milk 25 ml)
	Cereal 30 gm (e.g. Poha^1^)
Mid Morning:	Fruit 1 (100 gm)
Lunch:	Cereal + Starch Flour 120 gm (e.g. Rice Flour 60 gm + Arrowroot Flour 60 gm^2^) Vegetable 300 gm (e.g. Suran Vegetable^3^ + Dudhi Aloo Vegetable^4^)
	Lentil 10 gm (e.g. Thin Dal^5^) OR Yoghurt (Cow Milk based) 50 gm
Teatime:	Fruit 1 (100 gm)
Dinner:	Cereal + Starch Flour 120 gm (e.g. Rice Flour 60 gm + Arrowroot 60 gm)
	Vegetable 300 gm (e.g. Arbi Vegetable^6^+ Beans Potato Vegetable^7^)
	Lentil 10 gm (e.g. Thin Dal) OR Yoghurt (Cow Milk based) 50 gm
Note:	To use Oil and Ghee 6 tsp/day

English names: 1 = Indian Savour – Rice Flakes Based, 2 = Indian Bread, 3 = Root Vegetable, 4 = Bottle Gourd and Potato Vegetable, 5 = Lentil, 6 = Root Vegetable, 7 = Beans and Potato Vegetable

In any case, before advising dietary protein restriction, it is important to ensure that the appetite is normal. If the appetite is poor, there is no point advising LPD. Often, out of fear of dialysis, patients may not complain of poor appetite. In these cases, if there is worsening nutritional status as judged by weight loss, LPD should not be advised.

## Conclusions

The role of LPD in retarding progression of CKD is well established in animal experimental studies. Even in human subjects, there is enough evidence to suggest that LPD retards progression of CKD in carefully selected subjects. It should be prescribed to those with slowly progressive CKD at an early stage (stage 1–3) when the appetite and nutritional status are good, rather than at late stage (stage 4 & 5). It can be prescribed even at late stages of CKD if the appetite and nutritional status are good. It should not be prescribed to those with poor appetite and weight loss.

## Abbreviations

CKD: Chronic kidney disease
CrCl: Creatinine clearance
DCCT: Diabetes control and complication trial
ESRD: End stage renal disease
GFR: Glomerular filtration rate
KA: Ketoanalogues
LPD: Low protein diet
SNGFR: Single nephron glomerular filtration rate
VLPD: Very low protein diet
